# Patient experience of spinal immobilisation after trauma

**DOI:** 10.1186/s13049-019-0647-x

**Published:** 2019-07-22

**Authors:** Camilla Ikast Ottosen, Jacob Steinmetz, Mo Haslund Larsen, Josefine S. Baekgaard, Lars S. Rasmussen

**Affiliations:** 1 Department of Anaesthesia, Section 4231, Centre of Head and Orthopaedics, Rigshospitalet, University of Copenhagen, Juliane Maries Vej 10, DK-2100 Copenhagen, Denmark; 2 Trauma Centre, Centre of Head and Orthopaedics, Rigshospitalet, University of Copenhagen, Copenhagen, Denmark

## Abstract

**Background:**

Spinal immobilisation of blunt trauma victims with potential spinal cord injury is considered standard of care. The traditional management has, however, been increasingly questioned and concerns about harm have been raised. Few studies have described the perspective of the trauma patient regarding the spinal immobilisation.

The objective of this study was therefore to evaluate the patient experience of immobilisation after trauma.

**Methods:**

We prospectively screened adult trauma patients admitted to a level 1 trauma centre for eligibility. We included adult trauma patients who had been, and remembered being, immobilised for spinal protection with a cervical collar and a spine board prehospitally or upon arrival at the trauma centre. A semi-structured interview was conducted 2 to 72 h after admission either in person or by telephone.

**Results:**

One hundred and fourteen patients were eligible for inclusion based on the patient charts. Out of 98 patients assessed for participation, 48 (49%) had no memory of being immobilised.

We thus included 50 patients with a median age of 37 years (IQR: 26–60) of whom 38 (76%) were men. The median injury severity score was 9 (IQR: 3–15) and the median time with a cervical collar from initial application to in-hospital removal or until the interview was given was 91 min (IQR: 72–136).

Nineteen patients (38%) reported discomfort and 12 patients (24%) experienced pain related to the immobilisation. Forty patients (80%) reported a sense of protection related to the immobilisation.

**Conclusion:**

Discomfort related to spinal immobilisation was reported in 38% of trauma patients. However, a sense of protection was a recurring theme in 80% of the trauma patients, who recalled being immobilised. Nearly half of the awake trauma patients had no memory of being immobilised.

## Background

Spinal immobilisation of blunt trauma victims with potential spinal cord injury has been considered standard of care for several decades. Inadequate management of spinal injury may cause neurological deficits and spinal stabilisation has therefore been considered crucial for preventing such secondary injuries [1, 2].

Many researchers have, however, raised concerns about immobilisation and questioned its efficacy, the risk of over-triage and potential harmful effects as the evidence of the benefits of spinal immobilisation has been limited [1, 2]. Furthermore, several studies have identified complications related to immobilisation, such as tissue ischemia, pressure ulcers and decreased lung volumes [3–5]. These complications have, however, been measured objectively without accounting for the subjective experience secondary thereto, such as possible pressure, discomfort and dyspnoea related to being immobilised.

Hence, patients’ perspective regarding spinal immobilisation does not seem to have been evaluated.

The objective of this study was therefore to evaluate the patient experience of spinal immobilisation following trauma through a semi-structured interview.

## Methods

We conducted a single-centre, semi-structured interview study of patients, who had been immobilised for spinal protection with a cervical collar and a spine board after trauma. Patient informed consent was obtained prior to inclusion. The Danish Data Protection Agency approved the data management. Approval by the Committee on Health Research Ethics was not necessary according to Danish law.

### Participant selection

We prospectively screened trauma patients above 18 years of age admitted to the trauma centre at Rigshospitalet (RH), Copenhagen, a level one trauma centre receiving trauma patients from all parts of the eastern region of Denmark either by ambulance or helicopter, for eligibility.

We included adult trauma patients who had been immobilised for spinal protection with a cervical collar and a spine board prehospitally or upon arrival at the trauma centre. Only awake and alert trauma patients, who recalled being immobilised and were able to speak Danish, were included. Inclusion took place two to 72 h after admission and only after full spinal immobilisation was terminated, although neck collar was tolerated.

Patients, who were incompetent or presented with an acute psychiatric disorder, were excluded.

The ambulance personnel at scene, which includes a paramedic and their assistant, carried out the immobilisation of the trauma patients. In addition, in most cases a physician staffed mobile emergency care unit or a helicopter was also involved in the prehospital treatment of the patient [6].

Primarily, the patients were immobilised prehospitally with a spine board, cervical collar and head blocks. Upon arrival at the trauma centre, they were in most cases transferred from the spine board to a Trauma Transfer – still wearing the cervical collar and logrolled. In some cases, patients were on a scoop stretcher or a vacuum mattress and then transferred to the Trauma Transfer upon arrival.

In case the extent of immobilisation could not be clearly determined based on the patient charts, confirmation of immobilisation was sought through a detailed description by the patient if possible.

### Data collection and analysis

The interview guide was developed based on a review of studies on potential complications and disadvantages related to immobilisation with a cervical collar and a spine board as well as through a discussion among the research team. The interview covered the following topics: general experiences of being immobilised with a cervical collar and a spine board including experiences with the application of the immobilisation, knowledge of the use of immobilisation and experiences of disadvantages related to being immobilised.

The interviews were conducted two to 72 h after admission either in person or by telephone. Interviews were transcribed as close to verbatim as possible to a paper edition of the case report form during the interview and subsequently transferred to an electronic version of the case report form in Research Electronic Data Capture (REDCap) for later analysis [7].

Patient demographics were obtained through patient charts. In case the exact time of cervical collar application/removal was missing, total time with cervical collar was calculated from time points defined as halfway through treatment on-scene to halfway through treatment in the trauma bay. If the collar was not removed in the trauma bay, total time with cervical collar was defined from application to time of interview.

### Statistics

Characteristics were reported by giving medians with interquartile ranges (IQR), frequencies and percentages, while the interviews were reported by giving frequencies and percentages with 95% confidence intervals (CI) of specific statements. A two-sided t-test was used to test any significance between age and injury severity score (ISS), respectively, and the reporting of discomfort related to being immobilised.

#### Sample size

We estimated that if no more than six out of 50 patients experienced discomfort related to being immobilised, then the true proportion of discomfort would be 30% or less  based on the two-sided 95% confidence interval.

## Results

We screened 358 patients between April 14th, 2018, and August 31st, 2018, with an end of follow-up on September 30th, 2018.

A total of 114 trauma patients were eligible for inclusion based on the patient charts.

**Fig. 1:**
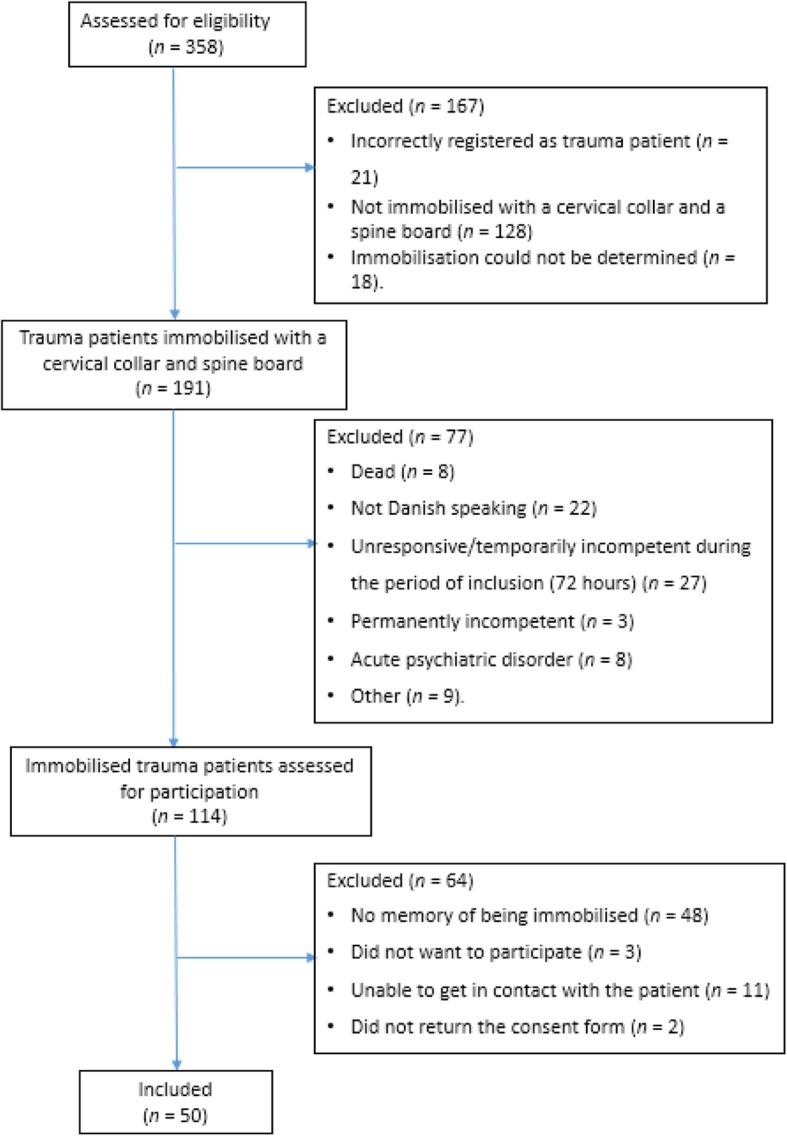
Flowchart of the screening process of eligible trauma patients

We were unable to get in contact with eleven patients, three patients refused to participate and two did not return the consent form and were therefore excluded. Out of the remaining 98 patients, otherwise eligible for inclusion, 48 (49%) had no memory of being immobilised (Fig. 1).

We included 50 patients with a median age of 37 years (IQR: 26–60) of whom the majority were men (76%). The most common trauma mechanism was trauma due to motor vehicle/−cycle collisions (28%) followed by bicycle accidents and falls from height (both 24%). The median injury severity score was 9 (IQR: 3–15) and 13 patients (26%) had an ISS above 15. Seven patients (14%) had a history of back pain based on the patient charts and another seven patients (16%) were obese with a BMI above 30 kg/m^2^. Data on BMI was missing for five patients (10%).

The median time with a cervical collar from initial application to in-hospital removal or until the interview was given was 91 min (IQR: 72–136) (Table 1).

**Table 1:** Characteristics of trauma patients immobilised with a cervical collar and a spine board (*n* = 50)

Gender, n (%)	
Male	38 (76)
Age (years), median [IQR*]	37[26–60]
BMI** (kg/m^2^), median [IQR*]	25 [23–28]
Missing	5
Significant comorbidities, n (%)	
History of back pain	7 (14)
Obesity (BMI** > 30 kg/m2)	7 (16)
Trauma mechanism, n (%)	
MVC/MCC^***^	14 (28)
Bicycle crash/collision	12 (24)
Fall from height	12 (24)
Other	12 (24)
ISS^****^, median [IQR*]	9 [3–15]
ISS^****^ > 15, n (%)	13 (26)
Time with cervical collar (minutes), median [IQR*]	91 [72–136]

* Interquartile range

** Body Mass Index

***Motor vehicle crash/motorcycle crash

****Injury Severity Score

The interviews were primarily conducted at the hospital 45 (90%) while 5 (10%) were conducted by telephone. Eight patients (16%) were still wearing the cervical collar at the time of the interview.

Nineteen patients (38%) reported discomfort and 19 patients (38%) experienced point pressure from the immobilisation, primarily at the hip (21%) and at the back of the head (16%).

Twelve patients (24%) experienced pain related to the immobilisation with no predominant location, mainly depending on the type of injury.

A sense of protection related to the immobilisation was reported in 40 patients (80%), mainly because they felt taken care of and that no further injury would occur.

Anxiety and dyspnoea related to the immobilisation were reported in three cases each (6%). Twenty-nine patients (58%) felt informed about the rationale for the immobilisation and 4 (8%) experienced that the staff had difficulties with the application of the immobilisation (Table 2).

**Table 2:** Results of the semi-structured interview of trauma patients’ experience of being immobilised with a cervical collar and a spine board (*n* = 50)

Experience of difficulty with the application of the immobilisation, n (%)	
*Yes*	4 (8)
*No*	40 (80)
*Do not know*	6 (12)
Felt informed about the reason for applying the immobilisation, n (%)	
*Yes*	29 (58)
*No*	13 (26)
*Do not know*	8 (16)
Pain related to the immobilisation, n (%)	
*Yes*	12 (24), 95% CI^*^ (14 to 38)
*No*	34 (68)
*Do not know*	8 (8)
Discomfort with the immobilisation, n (%)	
*Yes*	19 (38), 95% CI* (25 to 53)
Anxiety related to the immobilisation, n (%)	
*Yes*	3 (6)
Immobilisation providing a sense of protection, n (%)	
*Yes*	40 (80), 95% CI* (66 to 89)
Dyspnoea related to the immobilisation, n (%)	
*Yes*	3 (6)
*No*	46 (92)
*Do not know*	1 (2)
Pressure from the immobilisation, n (%)	
*Yes*	19 (38), 95% CI* (5 to 53)
*No*	29 (58)
*Do not know*	2 (4)

*Confidence interval

One patient stated that the immobilisation was one of the worst experiences ever.

The majority were aware of the reasoning for using immobilisation after trauma with a perception of protection of the spine and to avoid further damage.

The median ISS was lower amongst patients who experienced discomfort in relation to the immobilisation compared to patients who did not experience discomfort (5 (IQR: 2–12) versus 9 (IQR: 5–17)). There was, however, no significant difference between the two groups (*p*-value 0.13).

There was no significant difference in age according to reporting of discomfort with a median age of 43 years (IQR: 21–58) in the group who reported discomfort and a median age of 35 years (IQR: 28–59) for patients, who did not experience discomfort in relation to being immobilised, (*p*-value 0.67).

There were two cases of protocol violation, as the interviews were conducted approximately 84 h and one week after injury, respectively.

## Discussion

In this semi-structured interview, we found that nearly half of the awake trauma patients, otherwise eligible, had no memory of being immobilised. Discomfort related to immobilisation was reported in 38% of the trauma patients, while 80% felt a sense of protection related to being immobilised.

### Strengths and limitations

The strengths of this study include the prospective design with a detailed questionnaire. Furthermore, we had a large participation rate and inclusion within 72 h of admission to ensure that the patients would have a valid recall of the immobilisation.

Our study also has some limitations. The study was single-centred and based on a selected population of patients suspected of having severe injuries. This may have reduced the generalizability of our findings, as it could be argued that the possible seriousness of the trauma could have overshadowed the discomfort and even have induced a sense of protection related to being immobilised. Furthermore, nearly half of the patients had no memory of being immobilised, which could be contributed to the selected trauma population.

Our direct access to a CT scanner in the trauma bay allowed for quick clarification of whether the immobilisation was still required. The duration of the immobilisation could therefore be shorter than at other facilities and thus may have affected our findings as one could imagine the reporting of discomfort, pain and pressure related to the immobilisation could increase with the time being immobilised.

We also limited our study by only including patients who were able to recall being immobilised. Even though they did not remember the immobilisation, they could have experienced discomfort or anxiety during the time being immobilised, being potential cases of denial.

Furthermore, we cannot exclude that some of the patients, with no memory of the immobilisation, later would have been able to recall the immobilisation.

In case the actual immobilisation could not be clearly determined based on the patient charts, the patients in question were asked for a description of their immobilisation if possible to ensure eligibility. This could have led to inclusion of patients who did not fulfil the inclusion criteria of being fully immobilised with a cervical collar and a spine board, although we find this risk minimal.

Furthermore, there were two protocols violations, as interviews were not conducted within the predetermined timeframe. The effect of these, if any, is thought to be marginal.

Finally, when conducting an interview there is a risk that the interviewer affects or influences the answers depending on their questioning techniques. Negative experiences could be underreported when health care personnel interview patients. Furthermore, when asking about specific topics, the examiner may influence the patient’s response and thereby provoke answers on topics they had not even thought of themselves.

One should also remember that the experiences of the immobilisation represent a snapshot present at the time of the interview, as these could fluctuate or change over time.

Spinal immobilisation is a routinely performed procedure of blunt trauma victims with suspected spinal cord injury and has been considered crucial for preventing secondary injuries such as neurological deterioration [1, 8]. Despite spinal immobilisation being one of the most frequently performed prehospital interventions, high-level evidence demonstrating beneficial effects is lacking [1]. Thus, a systematic review from 2016 could not identify any instances of neurological deterioration among spine injured patients not immobilised in the prehospital environment, [9] hence the procedure primarily seems founded upon expert opinion rather than definitive evidence [8, 10–12].

In contrast, there is strong evidence that the prehospital spinal immobilisation is associated with complications ranging from discomfort to significant physiological compromise [13].

Spinal immobilisation with a neck collar may lead to airway management difficulty and therefore delay tracheal intubation or increase the risk of pulmonary aspiration [1, 2, 5, 11, 13]. The insertion of a central venous catheter can also be much more difficult.

Furthermore, one study found that application of a cervical collar causes a significant decrease in lung capacity and spirometry parameters, [3] but they did not examine the consequences hereof, such as whether this caused a subjective experience of dyspnoea.

Spinal stabilisation has also been associated with tissue ischemia and even an increased risk of pressure ulcers with prolonged use, [4] as well as an increased intracranial pressure [14–16].

Surprisingly, no studies seem to exist on the patient experience of spinal immobilisation after trauma. We found one study that compared different cervical collars in terms of patient comfort, but not the overall experience of being immobilised, [15] and another study, based on healthy volunteers with no prior history of back pain, compared spinal immobilisation with a backboard to a vacuum mattress-splint with respect to the incidence of symptoms generated by the immobilisation process. After being immobilised for 30 min, they found that standard backboard immobilisation was associated with an increased incidence and severity of occipital and lumbosacral pain [17].

The possible complications related to being immobilised were not determined in our study. There were, however, patients who experienced pressure and pain from the immobilisation, but only three patients (6%) experienced dyspnoea related to being immobilised.

Discomfort and pressure are related to being immobilised and these symptoms were more commonly detected than we anticipated. However, a sense of protection was a recurring theme in 80% of the trauma patients. The sense of protection primarily seemed to be based upon the reasoning for using the immobilisation, namely protect the spine and avoid further injury. Hence, if we move toward a more selective approach in the future, as the evidence of the beneficial effects of spinal immobilisation is lacking, we may have to take the patients current beliefs regarding spinal immobilisation into account.

As our study was based on a selective population of trauma patients, future research on patients not admitted to a level-one trauma centre could be of importance to enhance the knowledge of patient experience of spinal immobilisation after trauma.

## Conclusion

In conclusion, nearly half of the awake trauma patients had no memory of being immobilised. Discomfort related to spinal immobilisation was reported in 38% of trauma patients. However, a sense of protection was a recurring theme in 80% of the trauma patients, who recalled being immobilised.

## Data Availability

The dataset used and/or analysed during the current study are available from the corresponding author on reasonable request.

## Abbrevations

BMI: Body Mass Index
CI: Confidence interval
IQR: Interquartile range
ISS: Injury Severity Score
MCC: Motorcycle crash
MVC: Motor vehicle crash
REDCap: Research Electronic Data Capture
RH: Rigshospitalet
